# Mount Vernon Hospital

**Published:** 1913-08-09

**Authors:** J. Mackenzie

**Affiliations:** 133 Harley Street, W.


*To the Editor of* The Hospital.

### Mount Vernon Hospital.

*To the Editor of* The Hospital.

Sir,—Referring to your article on the Mount Vernon Hospital dispute in your issue of July 19, I beg to point out that I retired from the staff of the Mount Vernon Hospital some twelve months ago, and, in consequence of this, I have no knowledge of the matter under discussion, and certainly hold no opinion either one way or the other.—Yours faithfully,

J. Mackenzie.

133 Harley Street, W.

[We mentioned that Dr. Mackenzie's name was not included on the list of signatories to the document issued by the dissenting members of the hospital's staff.—Ed. The Hospital.]

